# Humoral Responses to Diverse Autoimmune Disease-Associated Antigens in Multiple Sclerosis

**DOI:** 10.1371/journal.pone.0129503

**Published:** 2015-06-11

**Authors:** Kishore Malyavantham, Bianca Weinstock-Guttman, Lakshmanan Suresh, Robert Zivadinov, Thomas Shanahan, Darlene Badgett, Murali Ramanathan

**Affiliations:** 1 Immco Diagnostics, Buffalo, NY, United States of America; 2 Department of Neurology, State University of New York, Buffalo, NY, United States of America; 3 Buffalo Neuroimaging Analysis Center, Department of Neurology, State University of New York, Buffalo, NY, United States of America; 4 Department of Pharmaceutical Sciences, State University of New York, Buffalo, NY, United States of America; Medical University of Innsbruck, AUSTRIA

## Abstract

To compare frequencies of autoreactive antibody responses to endogenous disease-associated antigens in healthy controls (HC), relapsing and progressive MS and to assess their associations with clinical and MRI measures of MS disease progression.

**Methods:**

The study analyzed 969 serum samples from 315 HC, 411 relapsing remitting MS (RR-MS), 128 secondary progressive MS (SP-MS), 33 primary progressive MS (PP-MS) and 82 patients with other neurological diseases for autoantibodies against two putative MS antigens CSF114(Glc) and KIR4.1a and KIR4.1b and against 24 key endogenous antigens linked to diseases such as vasculitis, systemic sclerosis, rheumatoid arthritis, Sjogren’s syndrome, systemic lupus erythematosus, polymyositis, scleroderma, polymyositis, dermatomyositis, mixed connective tissue disease and primary biliary cirrhosis. Associations with disability and MRI measures of lesional injury and neurodegeneration were assessed.

**Results:**

The frequencies of anti-KIR4.1a and anti-KIR4.1b peptide IgG positivity were 9.8% and 11.4% in HC compared to 4.9% and 7.5% in RR-MS, 8.6% for both peptides in SP-MS and 6.1% for both peptides in PP-MS (*p* = 0.13 for KIR4.1a and *p* = 0.34 for KIR4.1b), respectively. Antibodies against CSF114(Glc), KIR4.1a and KIR4.1b peptides were not associated with MS compared to HC, or with MS disease progression. HLA DRB1*15:01 positivity and anti-Epstein Barr virus antibodies, which are MS risk factors, were not associated with these putative MS antibodies.

**Conclusions:**

Antibody responses to KIR4.1a and KIR4.1b peptides are not increased in MS compared to HC nor associated with MS disease progression. The frequencies of the diverse autoreactive antibodies investigated are similar in MS and HC.

## Introduction

A role for humoral responses and B cells in multiple sclerosis (MS) pathogenesis has been suspected since the discovery of oligoclonal bands nearly 50 years ago [1]. Analysis of oligoclonal bands in cerebrospinal fluid (CSF) is now well established as a paraclinical assessment criterion for MS diagnosis [2]. Evidence suggests that somatic hypermutation of immunoglobulin-M (IgM) chains in B cells and clonal expansion, which usually occurs in germinal centers, is frequent in the CSF milieu of MS patients [3]. The efficacy of rituximab and ocrelizumab, which are monoclonal antibodies that specifically target B cells, in the treatment MS have underscored the importance of B cells in MS pathogenesis.

Autoreactive B cells can also generate antigen specific autoantibodies in serum that have proven to be useful for diagnosing a range of autoimmune diseases and paraneoplastic neurological diseases. Neuromyelitis optica (NMO) is a demyelinating inflammatory CNS disease that has a predilection to affect the optic nerves and spinal cord and is included in the larger CNS inflammatory idiopathic demyelinating disease group [4]. It has a more severe outcome, involves B cell-mediated pathogenesis and importantly, it is associated with serum anti-aquaporin-4 antibodies (NMO IgG) [4]. The discovery of serum NMO IgG antibodies as diagnostic for NMO and its positive clinical/therapeutic impact has provided a fresh impetus for discovering autoantibody biomarkers with diagnostic value for MS. Antibodies against myelin proteins such as myelin basic protein and myelin oligodendrocyte protein have been extensively investigated in MS serum and in CSF. However, the diagnostic value of antibodies against myelin proteins has not been conclusively proven and these tests have not found clinical acceptance [5, 6].

More recently, there have been promising reports of autoantibodies against CSF114(Glc) [7], a synthetic glycopeptide, and against the KIR4.1 inwardly-rectifying potassium channel [8] as having diagnostic value in MS. CSF114(Glc) autoantibodies recognized myelin and oligodendrocyte antigens in immunohistochemistry tests and were associated with development of brain lesions [7]. KIR4.1 immunoreactivity was found attenuated on MS lesions [9] and KIR4.1 antibodies were more frequent in acquired pediatric CNS demyelinating disorders compared to controls [10]. There is active interest in independently validating these novel serum antibodies as potential diagnostics for the unmet needs in MS.

There is also increasing interest in the role of comorbidities in MS progression [11, 12]. For example, elevated serum total cholesterol was shown to be associated with increased disability and brain injury in MS as assessed with quantitative MRI metrics [13, 14]. Comorbidities can delay diagnosis and treatment of MS and the timely identification and treatment of comorbidities could have a positive impact on MS disease course patient quality of life [11]. Autoimmune comorbidities that are eloquent outside the CNS could theoretically arise as a consequence of epitope spreading from the MS disease process or *vice versa*.

Epstein-Barr virus (EBV) is a common human herpes virus that causes infectious mononucleosis. A positive history of mononucleosis and high levels of anti-EBV antibodies are associated with increased MS risk and with greater clinical and MRI measures of MS disease progression [15–17]. Because EBV targets and establishes a chronic and lifelong infection of B cells, the resultant dysimmunity in the B cell compartment can potentially trigger autoreactive antibody responses.

The goals of our study were to systematically investigate the frequency, clinical and MRI associations of three recently proposed, putative MS-associated antibodies, CSF114(Glc), KIR4.1a and KIR4.1b peptides, and also a diverse range of autoantibodies linked to the other non-CNS autoimmune diseases in a large cohort of MS patients. We also investigated the associations of anti-EBV antibodies and HLA DRB1*15:*01* status with putative MS antigens and autoreactive antibodies because these are important risk factors in MS.

## Methods

### Study Population

#### Study Design

The study samples and MRI were obtained from an ongoing, prospective longitudinal study of clinical, genetic and environmental risk factors in MS at the MS Center of the State University of New York at Buffalo.

The University at Buffalo Human Subjects Institutional Review Board approved the study protocol and consent procedure. All participants provided written informed consent.

For this study, we analyzed 969 serum samples from 315 healthy controls, 411 relapsing remitting MS (RR-MS), 128 secondary progressive MS (SP-MS), 33 primary progressive MS (PP-MS) and 82 patients with other neurological diseases (OND). The percentages of neurodegenerative, vascular, autoimmune and neuromuscular categories of other neurological disease (OND) were 39%, 15%, 31% and 15%, respectively. The OND group contained a diverse group of diseases: the most common OND was Parkinson’s disease (14 patients) followed by migraines (8 patients), anti-phospholipid antibodies (7 cases), neuropathies (4 cases), myelopathies (3 cases), 2 cases each of Hashimoto’s encephalitis, Chiari malformation, mitochondrial disease, disc disease, acute disseminated encephalomyelitis, and vertigo. All subjects were recruited at the same center and with the same protocol. Serum samples were obtained within 3 hours of collection and stored at -80°C until use.

Patients and controls underwent neurological and MRI examinations and provided blood samples.

### MRI Acquisition and Analysis

MRI methods are summarized in S1 Methods. We used the T2 and T1-lesion volume (LV), and normalized whole brain volume (WBV) and gray matter volume (GMV) measures.

### Antibody Assays

The research scientists conducting analyses of antibodies were blinded to the patients’ clinical status. To assure high level of technical expertise, antibody assays against all autoantigens, including the KIR4.1 peptide antibodies assays, were conducted at Immco Diagnostics (Buffalo, NY), a CLIA accredited, ISO 9001:2008 certified, and FDA approved laboratory.

#### Anti-CSF114(Glc) Antibodies

A limited number of CSF114(Glc)-IgG and IgM ELISA kits were provided by Diesse Ricerche Srl, Italy. Specific immunoglobulins in the samples were allowed to bind to immobilized synthetic glucosylated peptides followed by detection using anti-human immunoglobulins (anti IgG or anti IgM) conjugated to horseradish peroxidase (HRP) and 3,3′,5,5′-tetramethylbenzidine (TMB) substrate provided by the kit. Assays were performed according to manufacturer’s recommended protocol for 600 consecutive subjects in a blinded manner.

In brief, 100 μl of 1:101 diluted samples were dispensed into 96-well plates along with controls and calibrators provided with the kit. Plates were incubated for 45 minutes at 37°C. Four washes were performed with the provided wash buffer before dispensing the provided enzyme-secondary antibody conjugate into each well. After a 45-minute incubation with conjugate and four wash steps, 100 μl of TMB substrate was added to each well of the plates. After a 15-minute incubation with substrate, the reaction was stopped with the provided reagent and colorimetric reactions were photometrically read at 450 nm.

#### Anti-KIR4.1 Antibodies

KIR4.1 peptide sequences from the first and second extracellular loop and contiguous intra-membrane regions previously identified and described by Hemmer and colleagues [16]. Peptide KIR4.1A (sequence: *N* terminus–GVVWYLVAVAHGDLLELDPPANHTPCVVQVHTLTGAFL–*C* terminus) consisted of amino acids 83–120 from the first and second extracellular loops of KIR4.1. Peptide KIR4.1B (sequence: *N* terminus–TIGYGFRYISEECPLAIVLLI–*C* terminus) consisted of amino acids 128–148 from the intra-membrane region adjacent to KIR4.1A. The peptides were custom ordered with N-terminal biotinylation from the same vendor as by Hemmer and colleagues [16] (JPT Peptide Technologies Inc., Germany).

The assays for KIR4.1A and KIR4.1B antibodies were developed and optimized by Immco Diagnostics (Amherst, NY). Nunc Maxisorp plates (ThermoFisher, Waltham, MA, USA) were coated with optimal levels (5 μg/ml) of recombinant streptavidin (ProSpec-Tany Technogene Ltd., NJ) followed by coating with biotinylated KIR4.1A or KIR4.1B peptides (at 2 μg/ml). Unbound peptides were washed and plates were blocked using immunoassay blocker (StabilCoat, Kem-En-Tec Diagnostics, Denmark) and stabilizer before incubation with study samples.

KIR4.1A or KIR4.1B peptide-coated plates were incubated with human sera (disease or control groups) diluted 100-fold for 1 hour followed by wash and incubations with anti-human IgG-HRP conjugate (anti-IgG or anti-IgM, both from Jackson Immunoresearch, West Grove, PA) and TMB substrate (Immco Diagnostics, Buffalo, New York). The concentrations of anti-human IgG and IgM conjugated to HRP were optimized using a commercially sourced panel of 64 normal human sera (ProMedDx LLC, MA) to obtain 95% or greater specificity.

To validate the streptavidin coating procedure, the streptavidin-coated ELISA plates were reacted with a biotin-labeled mouse monoclonal antibody, washed and blocked. The plate was incubated with streptavidin-HRP as a reporter, washing and addition of TMB substrate. A control plate coated with StabilCoat was processed in parallel in the same way.

#### Anti-Oxidized LDL, Cardiolipins, Rheumatoid Factor, and Anti-Nuclear Antibody Immunoassays

Study samples including the controls were screened using analyte-specific ELISA’s for anti-oxidized LDL (Ox-LDL, ImmuLisa Oxidized Low Density Lipoprotein Antibody Enhanced ELISA, Immco Diagnostics, Catalog number 5158), anti-cardiolipin antibody (ACA) screen, and rheumatoid factor (RF, ImmuLisa Rheumatoid Factor Screen Enhanced ELISA, Immco Diagnostics Catalog number 5138S). Specimen positive on ACA screen were further tested on isotype-specific ACA ELISAs for IgG, IgA, and IgM (ImmuLisa Cardiolipin IgA, IgG and IgM Antibody Enhanced ELISAs Immco Diagnostics Catalog numbers 5118A, 5118G and 5118M, respectively). According to the manufacturer’s product inserts for the ELISAs, the expected frequency of positivity in control sera is 4% for RF, 1.2% for anti-ACA IgA, 3% for anti-ACA IgG, and 2.5% for the anti-ACA IgM.

All of the above assays provide a optimized sample diluent, pre-diluted enzyme conjugate, wash buffer, TMB substrate, enzyme stop solution, procedural controls (positive, negative controls and pre-diluted calibrators for constructing a standard curve) and use a harmonized assay protocol.

In brief, 100 μl of 1:101 diluted samples were dispensed into 96-well plate along with controls and calibrators provided with the kit. Plates were incubated with sample for 30 minutes at ambient temperature followed by three washes with the wash buffer provided. After a 30-minute incubation with enzyme-conjugate and three wash steps to remove unbound conjugate, 100 μl of TMB colorimetric enzyme substrate was added to the wells. The reaction was stopped after 30 minutes with stop solution and the resulting colorimetric reactions were read at 450 nm on an ELISA plate reader.

ImmcoStripe ANA-Advanced Line immunoassays (LIA) were used for the detection of anti-nuclear antibodies (ANA). The ANA-Advanced LIA panel selected comprised of 21 most common nuclear and cytoplasmic autoantigens prevalent in systemic autoimmune diseases and achieve >95% overall specificity.

ANA-Advanced LIA strips were placed in wells of the reaction tray and pre-blocked for 10 minutes with the provided sample diluent. Following the blocking, 1.5 ml of 1:101 diluted samples were dispensed into each well of the LIA reaction tray and incubated for 60 minutes with rocking. After incubation, the strips were washed three times for a total of 15 minutes with the wash buffer and incubated in the provided pre-diluted enzyme conjugate for 30 minutes with rocking. The wash steps were repeated to remove unbound enzyme conjugate followed by incubation with TMB substrate for 10 minutes to produce blue colored lines. Each LIA strip has a cut-off and procedural control lines that aid in the qualitative interpretation of test results for 21 autoantibodies per sample.

#### Anti-EBV and Anti-Cytomegalovirus (CMV) Antibodies

Enzyme-linked immunosorbent assay (ELISA) kits from Diamedix Corporation (Miami, FL) were used to quantify anti-CMV IgG, anti-EBV early nuclear antigen-1 (EBNA-1) and anti-EBV viral capsid antigen (VCA) IgG antibodies in serum as previously described [18].

For the anti-CMV, anti-EBV VCA and anti-EBV EBNA-1 assays, serum samples were diluted in sample buffer to 1:500, 1:2000 and 1:1000, respectively. 100 μl aliquots of diluted samples, standards, or controls were added to the antigen-coated wells and incubated (37°C for 60 minutes for anti-CMV, 30 minutes room temperature for anti-VCA and anti-EBNA) to allow binding. After discarding the contents, wells were washed three times with wash buffer and incubated with 100 μl of conjugate solution (37°C for 20 minutes for anti-CMV, 30 minutes room temperature for anti-VCA and anti-EBNA). After incubation, 100 μl of stop solution was added to the well. Absorbance was read at 450 nm against a reference reading at 600 nm. Serial dilutions of positive control samples provided with each kit were used to generate standard curves. The anti-CMV, EBNA-1 and VCA IgG levels were normalized to the manufacturer’s cut-off calibrator standard, which represents a sample that is just positive [18]. For some analyses, the anti-EBNA-1 and anti-VCA relative concentrations were categorized into quartiles. The anti-CMV antibody titers were distinctly bimodal and analyzed as a dichotomous positive status variable.

### Genotyping

HLA DRB1*15:*01* status was obtained by genotyping DNA from peripheral blood for *rs3135005*, a SNP strongly correlated with HLA DRB1*15:*01* status, using an allele discrimination method using the OpenArray platform (Applied Biosystems, Life Technologies, Foster City, CA).

### Data Analysis

SPSS (IBM Inc., Armonk, NY, version 19.0) statistical program was used. In view of the multiple tests, the Benjamini-Hochberg method was used to assess significance with a target false discovery rate of 0.05 [19]. The tables and results summarize the raw, unadjusted *p*-values for all variables. Adjusted *p*-values (*q*-values) are shown only for variables with unadjusted *p*-values ≤ 0.05.

The associations of EDSS and quantitative MRI measures with antibodies were assessed in the MS patient sub-groups. The analysis was limited to those antibodies for which continuous values in EU/ml were available (CSF114(Glc) IgG, CSF114 IgM, KIR4.1a IgG, KIR4.1a IgM, KIR4.1b IgG, KIR4.1b IgM, Ox-LDL) and those with 30 or more positive cases (anti-DNA antibody status) to reduce multiple testing. T2-LV and T1-LV were logarithm (base 10) transformed for regression analyses to reduce skew. The EDSS dependent variable was analyzed using ordinal regression. The MRI parameters of interest, T2-LV, T1-LV, GMV and WBV were analyzed individually as dependent variables with linear regression. All regression analyses included age, gender, type of MS (RR-MS, SP-MS or PP-MS) were predictors.

The associations of autoantibodies with HLA DR*15:*01* positive and CMV positive status, anti-EBV VCA and anti-EBV EBNA-1 quartiles were assessed individually as predictors appropriate regression analysis; all of these analyses include age, gender, disease status (healthy control, RR-MS, SP-MS, PP-MS or OND) as predictors. Linear regression was used for continuous variables and logistic regression for binary variable. The analysis was limited to those antibodies for which continuous values in EU/ml were available (CSF114 IgG, CSF114 IgM, KIR4.1a IgG, KIR4.1a IgM, KIR4.1b IgG, KIR4.1b IgM, Ox-LDL, RF Screen and ACA Screen) and those with 30 or more positive cases (anti-DNA antibody status), which were treated as dependent variables.

## Results

### Demographic and Clinical Characteristics

The demographic, clinical, MRI and treatment characteristics of the study subsets are summarized in Table 1. The age, gender ratio, disease durations, clinical disability (as measured by the EDSS), MRI measures and disease-modifying treatments are representative of a typical clinical sample. Table 2 shows the list of antigenic targets that were analyzed and their disease-associations.

**Table 1:** Demographic, clinical, MRI characteristics and current disease-modifying therapies.

Characteristic	HC	RR-MS & CIS	SP-MS & RR/SP-MS	PP-MS & PR-MS	OND	*p-*value
Sample Size *n*	315	411	128	33	82	–
% Female	61.9%	69.8%	74.2%	54.5%	69.5%	0.029
Age, years	44.1 ± 16	42.2 ± 12	54.2 ± 9.1	54.1 ± 6.4	45.0 ± 17	
Disease duration, years	–	10.6 ± 9.1	22.7 ± 11.1	15.4 ± 10.6	9.5 ± 8.8	
EDSS*	–	2.0 (1.5)	6.0 (2.0)	6.0 (3.0)	–	
T2-LV, cm^3^	–	10.5 ± 14	19.7 ± 18	17.0 ± 20	–	
T1-LV, cm^3^	–	2.2 ± 5.3	5.1 ± 7.4	2.2 ± 2.5	–	
Sample size	228	383	117	33	72	
Brain volume, cm^3^	1538 ± 92	1509 ± 93	1417 ± 78	1445 ± 86	1521 ± 103	
Gray matter volume, cm^3^	782 ± 64	757 ± 71	701 ± 59	717 ± 46	760 ± 88	
Interferon-beta-1a	–	37%	33%	17%	–	
Interferon-beta-1b	–	0.7%	–	–	–	
Glatiramer acetate	–	20%	23%	26%	–	
Natalizumab	–	14%	14%	–	–	
None^§^	–	25%	23%	51%	–	

* All continuous variables (age, disease duration, T2-LV, T1-LV) are mean ± standard deviation. For the ordinal EDSS, the median (inter-quartile range) are given.

^§^ The remainder received other treatments.

**Table 2:** Antigenic targets and disease associations of antibodies assessed.

Antigen	Description and Disease Association	References
CSF114(Glc) IgG, CSF114 IgM	Putative MS	[7]
KIR4.1a IgG, KIR4.1a IgM	Putative MS	[8, 10]
KIR4.1b IgG, KIR4.1b IgM	Putative MS	[8, 10]
Ox-LDL	Cardiovascular/systemic vasculitis and associated disorders	[38, 39]
Rheumatoid factor	Rheumatoid arthritis	[40]
ACA IgA, ACA IgG, ACA IgM	Vasculitis	[41]
PM-Scl 100, PM-Scl 75	Systemic sclerosis	[41]
Ro-52, Ro-60	SS, SLE, polymyositis, scleroderma, MCTD	[41]
Jo-1	Polymyositis	[41]
Ribo-P	SLE, scleroderma	[41]
Nucleosomes	SLE, MCTD, SS, polymyositis, scleroderma	[41]
DNA	SLE, MCTD, Scleroderma	[41]
Sm	SLE, MCTD	[41]
U1RNP-68, U1RNP-A, U1RNP-C	MCTD, SLE	[41]
La	SS, SLE, scleroderma	[41]
Scl-70	Scleroderma, MCTD	[41]
Centromere B	Scleroderma, SLE	[41]
PCNA	SLE	[42]
Mi-2	Myopathies, dermatomyositis	[43]
Ku, SRP-54	Myopathies	[43]
AMA-M2	Primary biliary cirrhosis (PBC)	[44]
DFS70	Negative association with systemic autoimmune disease; found in healthy specimens	[45, 46]

SS: Sjogren’s Syndrome, SLE: Systemic lupus erythematosus, LDL: Low-density lipoprotein, MCTD: Mixed Connective Tissue Disease (MCTD), PBC: Primary biliary cirrhosis.

### Putative MS-Antigens

We first examined titers and positivity for IgG and IgM antibodies against three putative MS-associated antigens, CSF114(Glc), KIR4.1a and KIR4.1b. The frequency of positivity and titers are summarized in Table 3 and Table 4, respectively. We did not find any association of these antibodies with MS or with MS disease course (Table 3, also S1 Table). The anti-KIR4.1a and anti-KIR4.1b frequencies are qualitatively consistent with those in recent reports [20, 21] but did not support the findings in the original report [8].

**Table 3:** Frequency of positivity for different antibodies in healthy controls (HC), relapsing-remitting MS (RR-MS, includes CIS) and secondary progressive-MS (SP-MS includes secondary progressive MS, relapsing-remitting/secondary progressive MS), primary progressive MS (PP-MS includes primary relapsing MS) and Other Neurological Diseases (OND).

Antigen	HC	RR-MS & CIS	SP-MS & RR/SP-MS	PP-MS & PR-MS	OND	*p-*value *	*q-value*
**Sample Size *n***	**202**	**224**	**76**	**22**	**38**	–	
CSF114 IgG	15 (7.4%)	20 (8.2%)	9 (10.6%)	0	4 (10.5%)	0.55	NS
CSF114 IgM	1 (0.5%)	1 (0.4%)	0	0	0	0.95	NS
**Sample Size *n***	**315**	**411**	**128**	**33**	**82**	–	
KIR4.1a IgG	31 (9.8%)	20 (4.9%)	11 (8.6%)	2 (6.1%)	7 (8.5%)	0.13	NS
KIR4.1a IgM	17 (5.4%)	19 (4.6%)	2 (1.6%)	0	3 (3.7%)	0.29	NS
KIR4.1b IgG	36 (11.4%)	31 (7.5%)	11 (8.6%)	2 (6.1%)	10 (12.2%)	0.34	NS
KIR4.1b IgM	17 (5.4%)	19 (4.6%)	2 (1.6%)	0	3 (3.7%)	0.29	
Ox-LDL	12 (3.8%)	13 (3.2%)	8 (6.2%)	2 (6.1%)	5 (6.1%)	0.46	NS
RF Screen	30 (9.5%)	37 (9.0%)	19 (14.8%)	3 (9.1%)	17 (20.7%)	0.014	0.22
ACA IgA	0	1 (0.2%)	2 (1.6%)	0	0	0.095	NS
ACA IgG	9 (2.9%)	11 (2.7%)	4 (3.1%)	0	2 (2.4%)	0.90	NS
ACA IgM	8 (2.5%)	10 (2.4%)	4 (3.1%)	0	3 (3.7%)	0.84	NS
ACA Screen	26 (8.3%)	29 (7.1%)	14 (10.9%)	0	8 (9.8%)	0.10	NS
PM-Scl 100	5 (1.6%)	10 (2.4%)	1 (0.8%)	0	3 (3.7%)	0.48	NS
PM-Scl 75	2 (0.6%)	7 (1.7%)	0	1 (3.0%)	0	0.22	NS
Ro-52	9 (2.9%)	14 (3.4%)	5 (3.9%)	2 (6.1%)	3 (3.7%)	0.89	NS
Ro-60	4 (1.3%)	2 (0.5%)	1 (0.8%)	0	5 (6.1%)	0.001	0.034
Jo-1	2 (0.6%)	1 (0.2%)	1 (0.8%)	0	1 (1.2%)	0.77	NS
Ribo-P	3 (1.0%)	2 (0.5%)	0	0	1 (1.2%)	0.71	NS
Nuclear	2 (0.6%)	4 (1.0%)	0	0	0	0.67	NS
DNA	21 (6.7%)	31 (7.5%)	4 (3.1%)	0	2 (2.4%)	0.098	NS
Sm	1 (0.3%)	1 (0.2%)	0	0	0	0.95	NS
U1-68	14 (4.4%)	11 (2.7%)	4 (3.1%)	2 (6.1%)	5 (6.1%)	0.46	NS
U1-A	9 (2.9%)	12 (2.9%)	1 (0.8%)	0	2 (2.4%)	0.58	NS
U1-C	10 (3.2%)	8 (1.9%)	3 (2.3%)	0	2 (2.4%)	0.74	NS
La	13 (4.1%)	10 (2.4%)	3 (2.3%)	1 (3.0%)	2 (2.4%)	0.72	NS
Scl-70	3 (1.0%)	1 (0.2%)	1 (0.8%)	0	0	0.64	NS
CENPB	7 (2.2%)	8 (1.9%)	3 (2.3%)	0	6 (7.3%)	0.053	NS
PCNA	0	0	0	0	0	–	
Mi-2	5 (1.6%)	2 (0.5%)	0	1 (3.0%)	1 (1.2%)	0.27	NS
Ku	1 (0.3%)	0	0	1 (3.0%)	1 (1.2%)	0.019	0.19
SRP-54	2 (0.6%)	2 (0.5%)	2 (1.6%)	0	1 (1.2%)	0.71	NS
AMA-M2	3 (1.0%)	4 (1.0%)	1 (0.8%)	1 (3.0%)	2 (2.4%)	0.62	NS
DFS70	3 (1.0%)	2 (0.5%)	0	1 (3.0%)	0	0.28	NS

* Pearson chi-square

**Table 4:** Antibody titers (EU/ml) expressed as median (25^th^ percentile– 75^th^ percentile). Anti-EBV-EBNA1 and anti-EBV VCA titers are expressed in normalized units.

Antigen	HC	RR-MS & CIS	SP-MS & RR/SP-MS	PP-MS & PR-MS	OND	*p-*value *	*q-value*
CSF114 IgG	3.2 (1.4, 8.0)	2.6 (1.2, 6.3)	2.4 (0.9, 6.9)	3.1 (1.3, 5.0)	2.1 (1.3, 5.2)	0.50	NS
CSF114 IgM	4.4 (2.7, 6.9)	4.1 (2.3, 6.7)	3.7 (2.0, 5.5)	4.1 (2.9, 6.0)	3.9 (2.5, 6.6)	0.32	NS
KIR4.1a IgG	12 (8.6, 16)	11 (7.3, 15)	12 (7.4, 16)	10 (6.7, 16)	12.8 (8.3, 17)	0.053	NS
KIR4.1a IgM	7.3 (4.2, 11)	6.5 (3.9, 11)	5.6 (3.2, 8.9)	5.6 (4.5, 10)	6.7 (3.8, 11)	0.030	0.098
KIR4.1b IgG	12.2 (9.0, 16)	11.4 (7.7, 15)	11.4 (7.6, 16)	10.6 (5.6, 17)	12.9 (7.9, 17)	0.059	NS
KIR4.1b IgM	7.0 (4.3, 11)	6.3 (3.8, 10)	5.4 (3.1, 9.2)	5.5 (4.3, 10)	6.7 (3.5, 11)	0.039	0.13
Ox-LDL	10.1 (8.2, 13)	9.5 (7.7, 12)	9.4 (7.3, 13)	8.5 (6.9, 11)	13.4 (10, 18)	0.069	NS
RF Screen	8.8 (6.0, 13)	10 (7.0, 14)	12.1 (8.1, 16)	13.1 (8.0, 17)	11.6 (7.9, 18)	< 0.001	< 0.001
ACA Screen	7.4 (4.5, 11)	7.0 (4.5, 11)	6.8 (4.3, 13)	6.7 (3.8, 8.7)	7.95 (4.8, 12)	0.49	NS
EBV EBNA1	27.4 (11, 69)	142 (53, 280)	85.8 (38, 181)	94.5 (33, 201)	36.2 (6.7, 110)	< 0.001	< 0.001
EBV VCA	38.7 (12, 81)	90.6 (38, 181)	106 (50, 229)	71.2 (37, 157)	58.1 (23, 104)	< 0.001	< 0.001

*Kruskal-Wallis test

### Putative Autoimmune-Antigens

We assessed the frequency of antibody responses against a wide range of antigens associated with autoimmune diseases using clinically validated assays (Table 3). The antigens assessed were diverse in their molecular characteristics (ranging from proteins, lipids and nucleic acids) and in their organ and cellular location. The highest frequencies of positive antibody responses were found for RF screen. RF titer levels were higher in the SP-MS and OND groups compared to the HC and RR-MS groups (*q* < 0.001 in Table 4, S1 Fig). Overall, the relative frequencies of positivity against the remaining autoimmune targets were low: only DNA and anti-ACA positive frequencies in MS were greater than 5%. We did not find evidence for associations with disease-associated antigens shown in Table 3 except for Ro-60 (*q* = 0.034). There were no differences between MS and controls, however, because the differences in Ro-60 frequencies were caused by the higher frequencies in the other neurological disease group. Table 4 also summarizes antibody titers for Ox-LDL and ACA screen antigens.

These results are consistent with the conclusion that autoimmune co-morbidities with which the antigenic targets in Table 3 are linked to are infrequent in MS.

### Clinical and MRI Associations

The clinical and MRI associations were limited to the MS subsets and to the autoantibodies for which we had standardized titers (CSF114(Glc) IgG, CSF114(Glc) IgM, KIR4.1a IgG, KIR4.1a IgM, KIR4.1b IgG, KIR4.1b IgM, Ox-LDL and RF Screen) and to anti-DNA positivity status, the only antigen that had sufficient number (*n* ≥ 30) or more positive MS subjects. There were no associations with EDSS or any of the lesional (T2-LV and T1-LV) and neurodegeneration (WBV and GMV) MRI measures for any of the antibodies in Table 5.

**Table 5:** Associations with MRI and clinical measures.

Antigen	EDSS	T2-LV	T1-LV	GMV	WBV
CSF114 IgG	0.057	0.72	0.48	0.79	0.29
CSF114 IgM	0.015 (0.15)	0.30	0.95	0.67	0.52
KIR4.1a IgG	0.14	0.63	0.43	0.80	0.79
KIR4.1a IgM	0.33	0.73	0.36	0.93	0.35
KIR4.1b IgG	0.032 (0.16)	0.58	0.48	0.66	0.70
KIR4.1b IgM	0.41	0.83	0.40	0.86	0.47
Ox-LDL	0.13	0.16	0.80	0.97	0.86
RF Screen	0.17	0.25	0.43	0.013 (0.13)	0.90
ACA Screen	0.65	0.79	1.0	0.57	0.77
DNA	0.25	0.44	0.81	0.06	0.073

EDSS: Expanded disability status scale; T2-LV: T2 lesion volume; T1-LV: T1 lesion volume; GMV: Normalized gray matter volume; WBV: Normalized whole brain volume. EDSS was analyzed with ordinal regression and the MRI variables were analyzed with linear regression. All analyses corrected for age, gender and type of MS. The Table entries summarize the unadjusted *p*-values from regression analyses. The *q*-values are shown in parentheses for cells with *p* ≤ 0.05

### HLA DR*15:*01* Associations

HLA DR*15:*01* is the best-established genetic variation that has been associated with MS risk across multiple studies. The HLA DR*15:*01* positive frequency in MS patients was (52.8% in MS vs. 32.8% in healthy controls and 29.1% in OND). HLA DR*15:*01* was associated with anti-EBV EBNA-1 status and anti-VCA status (both *p* < 0.001). HLA DR*15:*01* positive status was not associated with titers or positivity status for the disease-associated antigens shown in Table 6.

**Table 6:** Associations with HLA DR*15:*01*, anti-EBV-EBNA-1 quartiles and anti-EBV-VCA quartiles and anti-CMV positivity.

Antigen	HLA*15:01 Status	Anti-EBV EBNA-1 Quartiles	Anti-EBV-VCA Quartiles	CMV Positivity
CSF114 IgG	0.41	0.21	0.28	0.29
CSF114 IgM	0.52	0.067	0.68	0.038 (0.19)
KIR4.1a IgG	0.092	0.74	0.12	0.047 (0.16)
KIR4.1a IgM	0.10	0.50	0.049	0.053
KIR4.1b IgG	0.076	0.60	0.29	0.072
KIR4.1b IgM	0.097	0.69	0.059	0.091
Ox-LDL	0.49	0.37	0.006 (0.06)	<0.001 (< 0.001)
RF Screen	0.097	0.097	0.79	0.14
ACA Screen	0.89	0.014 (0.14)	0.063	0.98
DNA*	0.73	0.19	0.33	0.68

* Anti-DNA positivity status was analyzed with logistic regression and the remaining titer variables were analyzed with linear regression. All analyses corrected for age, gender and type of MS.

The Table entries summarize the unadjusted *p*-values from regression analyses. The *q*-values are shown for cells with *p* ≤ 0.05.

### Anti-Virus Antibody Associations

The levels of anti-EBV EBNA-1 and anti-EBV VCA antigens were higher in each of the MS sub-groups compared to controls (Table 4). The *q*-values for the associations of anti-EBV EBNA-1 and anti-EBV VCA levels with antibodies against putative MS antigens and autoimmune antigens were not significant (Table 6). Anti-CMV positivity was associated with Ox-LDL titers (*q* < 0.001, Table 6).

## Discussion

In this study, we investigated the role of autoantibodies against three putative MS antigens (CSF114(Glc), KIR 4.1a and KIR 4.1b peptides) and 26 other disease-linked antigens using validated assays in a large cohort of 969 subjects.

Levels of IgM antibodies against CSF114(Glc) were shown to be significantly elevated in MS patients compared to blood donor controls or other autoimmune conditions [22]. Later the synthetic peptide was demonstrated to be a reliable and specific probe for prognosis and monitoring of MS [7]. We did not find evidence to support associations of the putative MS antibodies with MS (compared to controls). None of the other disease-linked antibodies were associated with disability or with the assessed MRI measures in MS patients.

Smoking has been reported to increase the risk for developing anti-interferon neutralizing antibodies [23, 24]. We had self-reported smoking status for 903 subjects. Ever-smoking status was associated with increased anti-EBV EBNA1 antibodies. However, we did not obtain evidence that smoking was associated with increased levels of putative MS antibodies or with other disease-linked antibodies.

We also had treatment information, which was summarized in Table 1. One limitation and potential criticism of our study is that we did not extensively investigate the effects of MS treatments. MS treatments, particularly those that target B cells, e.g., rituximab, ocrelizumab and azathioprine treatments, can affect humoral autoantibody responses directly. Other immunomodulatory treatments such as interferon beta and natalizumab may affect humoral autoantibody responses via their effects on other immune cells subsets that interact with B cells. We opted not to pursue sub-analyses of treatments because the overall frequency of autoantibody positivity was low and the impact on MRI measures was not statistically significant.

There have been multiple studies showing an increase in co-occurrence of different autoimmune diseases with MS [25–31]. However, in a recent study that included a large sample of MS patients (*n* = 20,276) and their parents (*n* = 23,290) there was no consistent increase in frequency of other autoimmune diseases co-occurring with MS [32]. Furthermore, the frequency of autoimmune diseases other than MS was not increased in first-degree relatives [32]. Although our study used autoantibodies, our findings were similar in that we did not find evidence to support an increased frequency of autoantibodies in MS compared to controls. The design of our study extends the findings from population-based studies to the tertiary clinical setting and we were able to systematically assess the associations of autoantibodies with MRI and clinical measures. The family-based study by Barcellos *et al*. [25] and the population-based study by Ramagopalan *et al*. [31] used patient self-reports whereas the Langer-Gould *et al*. population-based study [28] used electronic clinical records from an insurance and managed care provider to assess the presence of concurrent autoimmune disease. Solomon *et al*. [33] reported the prevalence of ANA to be 31%, extractable nuclear antigen or SSA antibodies to be 4%, rheumatoid factor to be 1.1%, anti-cardiolipin antibodies (IgG, IgM or IgA) to be 16%, and lupus anticoagulant (LA) to be 2.2% in a group of 91 female MS patients. Similar to our results, they did not find associations with disability on the self-reported Disability Status Scale [33]. However, the Solomon *et al*. [33] study did not include male MS patients or a control group. Notably, Eaton et al. used the ICD-10 codes from the comprehensive Danish National Hospital Registry and found that autoimmune diseases in aggregate were present in 4% of the general Danish population over a 30-year period. In contrast to these studies, we employed blinded laboratory-based assessment with a panel of validated autoantibody assays.

The key strengths of our study are its large sample size of 969 subjects, the breadth of autoantibodies that were investigated, and the extensive clinical and MRI data on our patients. We also included HLA DRB1*15:*01* positivity, anti-EBV and anti-CMV antibody data in our analysis.

Our results deviate markedly from the results of Srivastava *et al*. who found 186 of 397 (46.9%) of MS patients, 0.9% of OND and none of the 59 controls had anti-KIR 4.1 antibodies [8]. Our findings are qualitatively comparable to those reported by Nerrant *et al*. who reported finding KIR4.1 antibodies in 7.5% of MS patients, 4.3% of OND patients and 4.4% of healthy controls [20]. Brickshawana *et al*. found that only 3 of 286 MS sera and 2 of 208 serum samples from controls showed KIR4.1 reactivity [21]. In this paper, we assessed antibodies against both KIR4.1a and KIR4.1b. Nerrant *et al*. and Brickshawana *et al*. reported results only for the KIR4.1a peptide [20, 21]. The original report of KIR4.1 antibodies found that the KIR4.1a peptide was approximately 10-fold more sensitive than KIR4.1b peptide; KIR4.1a peptide was used for all the competition experiments [8]. Subsequently reports from the same group described use of capture ELISA with full-length KIR4.1 protein derived from transiently transfected human endothelial kidney HEK293 cells rather than KIR4.1a peptide [9, 10]. Therefore, our study can be potentially criticized because we focused on the KIR4.1a and KIR4.1b peptides reported to be diagnostic by Srivastava *et al*. rather than purified full length KIR4.1 protein. However, Brill *et al*. reported that higher frequency of antibodies against anti-KIR4.1 peptide 83–120 in MS compared to controls [34]. The frequency in the MS group was 26.3% (21 of 80) vs. 6.7% (2 of 30) in the control group, which are lower than the frequency reported by Srivastava *et al*. [8]. Surprisingly, the frequency of anti-KIR4.1 positivity in the MS group was similar to that in the NMO group (22%, 10 of 45 patients).

A recent review by Hemmer [35] that was published after this manuscript was submitted, described the methodological factors that could potentially explain the replication challenges described by reported by Nerrant *et al*. and Brickshawna *et al*. [20, 21]. Hemmer [35] reported that structure of the targeted domains of the KIR4.1 protein, loop conformation, tetramer formation, glycosylation and cellular context are all important technical considerations necessary to detect anti-KIR4.1 antibodies and can explain discrepant results reported by Nerrant *et al*. and Brickshawna *et al*. [20, 21]. The review indicates they incorporated hydrophobic amino acids and biotinylation-streptavidin interactions that could cause the conditions needed for tetramer formation on the ELISA plates and for cooperative binding of autoantibodies. The review also indicates that the peptide-based assay had lower sensitivity and was difficult to control [35]. The full length glycosylated KIR4.1 protein from transiently transfected HEK cells used in the original report [8] was found to differ from that in brain tissue in molecular weight [35]. Even in glial cells, differential KIR4.1 post-translational modifications such as glycosylations unique to oligodendrocytes but not astrocytes may be essential to obtain optimal recognition by disease-associated anti-sera [35, 36]. Our peptides were not glycosylated and we did not confirm tetramer formation or cooperative binding of antibodies on the ELISA via biophysical techniques and positive controls. Additionally, we did not have positive sera from MS patients. Therefore, the possibility that our peptide assay did not replicate the exact conditions of the original report despite our efforts to do so cannot be formally excluded.

Although the recent publications by Nerrant *et al*. and Brickshawana *et al*. [20, 21] reduce the novelty of our negative findings somewhat, our study was able to address important mechanistic questions related to the associations of KIR4.1 antibodies to MS genetic and environmental risk factors and to MS disease progression in a much larger study sample. The benefit to the broader scientific community is still high given that it remains unclear why there are discrepancies in the results; the current study does add to the knowledge base on this assay and its significance.

Filippi *et al*. provide an excellent discussion of the pros and cons of a peptide vs. native antigen for MS autoantibody diagnostic assays [37]. Careful comparison of the ELISA assays for anti-KIR4.1 peptide antibodies reveals the finer differences. The original Srivastava *et al*. study [8] employed a peptide ELISA with two biotinylated peptides that were theoretically expected to form loops on the plate surface to generate epitopes for binding of disease specific anti-KIR4.1 peptide antibodies. Brill *et al*. used non-biotinylated peptides but observed significant anti-KIR4.1 antibody positivity [34]. Interestingly, Brill *et al*. [34] used a serum dilution factor of a 1000, which requires high analytical detection sensitivity for the method. The methods used in our studies and Brickshawana *et al*. [21] were similar to the Srivastava *et al*. study [8]. We even ordered peptides from the same company (JPT peptide Technologies, Berlin, Germany).

We also examined the associations of the autoantibodies with other risk factors for MS susceptibility and progression including HLA DRB1*15:01 positivity, anti-EBV EBNA-1 and anti-EBV VCA antibodies and anti-CMV antibodies. The hypothesis that any putative MS autoantibody would also be associated with HLA DRB1*15:01 positivity has considerable face validity since HLA DRB1*15:01 increases the risk for MS 3–4 fold and because of the role of *HLA* loci in providing T cell help to B cells. HLA Class II molecules (and CD21) are also co-receptors for EBV. Anti-EBV antibodies are associated with increased MRI lesion activity and greater brain atrophy [15–18]. We found HLA DRB1*15:01 positivity was associated with anti-EBV VCA antibodies and anti-EBV EBNA1 antibodies (*p* < 0.001) but not with the putative MS antibodies.

The presence of autoantibodies was relatively infrequent in primary progressive MS compared to MS and healthy control groups. The exact reasons for this are not completely clear and it must be stressed that the sample size of our PPMS group was smaller than the other groups. However, this finding can be construed as consistent with the greater importance of neurodegenerative processes relative to inflammatory immune-mediated processes in PPMS.

In conclusion, our findings demonstrate that the autoantibodies examined are not more frequent in MS patients compared to healthy controls. The presence of autoreactive antibodies was not associated with clinical and MRI measures of MS disease progression. Our results also cast further doubt on the reports that have suggested that CSF114 and anti-KIR 4.1a and KIR 4.1b peptide antibodies are diagnostic for MS. Nonetheless, more research into putative MS autoantibodies is warranted given their proven track record and clinical diagnostic value in a diverse range of other autoimmune and infectious disease states.

## Supporting Information

S1 Methods MRI acquisition and analysis. (PDF)

S1 Fig Levels of rheumatoid factor antibodies in EU/ml in healthy controls (HC), relapsing-remitting (includes CIS), secondary-progressive MS (SP-MS), primary-progressive MS (PP-MS) and other neurological disease (OND) groups. The bars represent mean levels and the error bars are standard errors. The p-values are from a Mann-Whitney tests compared to the HC group.
(PDF)

S1 Table Frequency of positivity for different combinations of KIR4.1 antibodies. (PDF)
