# Supplementary material for: Humoral Responses to Diverse Autoimmune Disease-Associated Antigens in Multiple Sclerosis
Source: PLoS One. 2015 Jun 11;10(6):e0129503. doi: 10.1371/journal.pone.0129503 (PMC4466031; doi:10.1371/journal.pone.0129503)
Supplement: S1 Methods — (PDF) [file pone.0129503.s001.pdf]

Supplementary Methods

MRI Acquisition and Analysis

Image Acquisition: Patients underwent brain MRI on a 3-T General Electric Signa 4x/Lx, scanner. Axial dual fast spin-echo (FSE) T2/PD-weighted image (WI), 3D-spoiled-gradient recalled (SPGR) T1-WI, spin echo (SE) T1-WI with and without gadolinium (Gd) contrast, fast attenuated inversion recovery (FLAIR) scans were acquired.

Image Analysis: The MRI analysts were blinded to patients' clinical characteristics and clinical status. The following MRI measures were computed: T1-, T2- and gadolinium (Gd) contrast-enhancing (CE) lesion volumes (LV), measures of central, global and tissue specific brain atrophy.

Lesion Measures: T2- and T1-LVs were obtained with a semi-automated edge detection contouring-thresholding technique previously described [1].

Global and Central Atrophy Measures: The SIENAX cross-sectional software tool was used, with correction for T1-hypointensity misclassification, for brain extraction and tissue segmentation [2]. We acquired and used normalized volume measures of the whole brain (WBV), GM (GMV), white matter (WMV), and lateral ventricles (LVV), as described previously [3].

We used the T1-, T2-LV, whole brain WBV and GMV measures for statistical analyses.

References

1. Zivadinov R, Rudick RA, De Masi R, Nasuelli D, Ukmar M, et al. (2001) Effects of IV methylprednisolone on brain atrophy in relapsing-remitting MS. Neurology 57: 1239-1247.

2. Smith SM, Zhang Y, Jenkinson M, Chen J, Matthews PM, et al. (2002) Accurate, robust, and automated longitudinal and cross-sectional brain change analysis. Neuroimage 17: 479-489.

3. Zivadinov R, Weinstock-Guttman B, Benedict R, Tamano-Blanco M, Hussein S, et al. (2007) Preservation of gray matter volume in multiple sclerosis patients with the Met allele of the rs6265 (Val66Met) SNP of brain-derived neurotrophic factor. Hum Mol Genet 16: 2659-2668.
