# Supplementary material for: Humoral Responses to Diverse Autoimmune Disease-Associated Antigens in Multiple Sclerosis
Source: PLoS One. 2015 Jun 11;10(6):e0129503. doi: 10.1371/journal.pone.0129503 (PMC4466031; doi:10.1371/journal.pone.0129503)

Supplementary Figure

Supplementary Figure 1. Levels of rheumatoid factor antibodies in EU/ml in healthy controls (HC), relapsing-remitting (includes CIS), secondary-progressive MS (SP-MS), primary-progressive MS (PP-MS) and other neurological disease (OND) groups. The bars represent mean levels and the error bars are standard errors. The p-values are from a Mann-Whitney tests compared to the HC group.

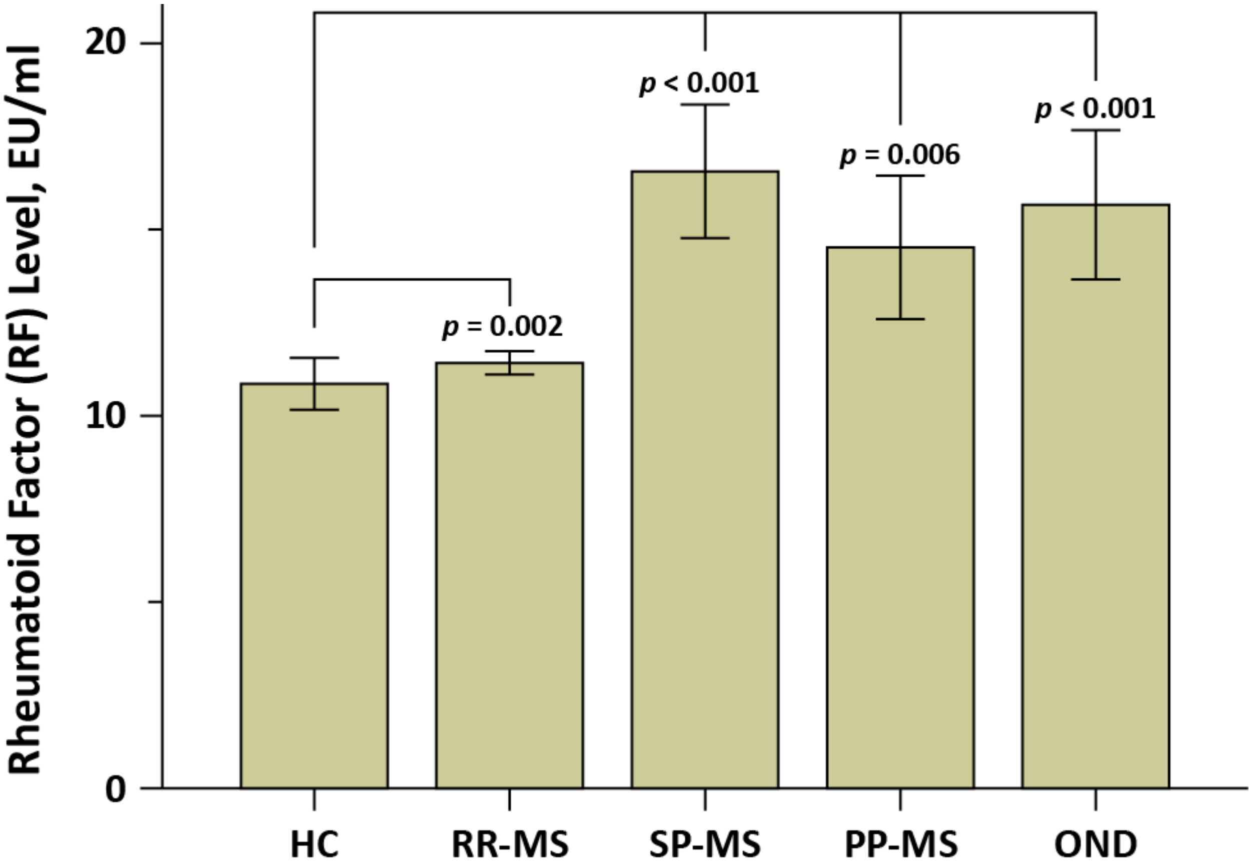

Supplement: S1 Fig — The bars represent mean levels and the error bars are standard errors. The p-values are from a Mann-Whitney tests compared to the HC group. (PDF) [file pone.0129503.s002.pdf]
