# Supplementary material for: Humoral Responses to Diverse Autoimmune Disease-Associated Antigens in Multiple Sclerosis
Source: PLoS One. 2015 Jun 11;10(6):e0129503. doi: 10.1371/journal.pone.0129503 (PMC4466031; doi:10.1371/journal.pone.0129503)
Supplement: S1 Table — (PDF) [file pone.0129503.s003.pdf]

Supplementary Table

Supplementary Table 1. Frequency of positivity for different combinations of KIR4.1 antibodies.

Antigen	HC	RR-MS & CIS	SP-MS & RR/SP-MS	PP-MS & PR-MS	OND	*p*-value *
**Sample Size *n***	**315**	**411**	**128**	**33**	**82**	–
Any KIR4.1 (KIR4.1a IgG OR KIR4.1a IgM OR KIR4.1b IgG OR KIR4.1b IgM)	61 (19.4%)	61 (14.8%)	18 (14.1%)	3 (9.1%)	14 (18.3%)	0.31
Total Positives (KIR4.1a IgG + KIR4.1a IgM + KIR4.1b IgG + KIR4.1b IgM)	1= 23 (7.3%)<br>2 = 36 (11%)<br>3 = 2 (0.6%)	1 = 33 (8.0%)<br>2 = 28 (6.8%)<br>3 = 0	1 = 10 (7.8%)<br>2 = 8 (6.3%)<br>3 = 0	1 = 2 (6.1%)<br>2 = 1 (3.0%)<br>3 = 0	1 = 7 (8.5%)<br>2 = 8 (9.8%)	0.45
Any IgG (KIR4.1a IgG OR KIR4.1b IgG)	45 (14.3%)	39 (9.5%)	16 (12.5%)	3 (9.1%)	12 (14.6%)	0.30
Any IgM (KIR4.1a IgM OR KIR4.1b IgM)	18 (5.7%)	22 (5.4%)	2 (1.6%)	0	3 (3.7%)	0.21
